# Strategic orchestration in sharing economies: A configurational analysis of platform differentiation and governance alignment

**DOI:** 10.1371/journal.pone.0326774

**Published:** 2025-06-25

**Authors:** Feifei Shao, Nianxin Wang

**Affiliations:** School of Economics and Management, Jiangsu University of Science and Technology, China; Fooyin University, TAIWAN

## Abstract

Platform firms in the digital economy face a critical challenge: balancing differentiation-driven competitive strategies with the need for institutional legitimacy. While existing research recognizes this tension, current frameworks inadequately address how governance mechanisms mediate strategic positioning in trust-sensitive sharing economy contexts. This study integrates Porterian positioning theory with platform boundary theory to analyze 33 chinese sharing platforms through fuzzy-set Qualitative Comparative Analysis (fsQCA), examining configurations of three external positioning dimensions (variety-based, gender-based, and region-based differentiation) and two internal governance dimensions (resource ownership and transaction process differentiation). Our analysis identifies two equifinal success pathways: (1) An aggressive strategy combining market pioneering in user segmentation with cautious transaction governance, and (2) a conservative strategy aligning geographic specialization with institutional compliance in resource ownership. Both pathways demonstrate that governance mechanisms compensate for the legitimacy risks inherent in differentiation strategies, with platform performance contingent on strategic coherence between external positioning and internal governance architectures. Three key theoretical advances emerge: First, integrating Porterian positioning theory with platform boundary theory, we demonstrate how governance dimensions serve as compensatory tools that enable radical market differentiation in sharing economy platforms without legitimacy erosion, a trade-off insufficiently addressed in prior distinctiveness theory research. Second, building on configuration theory, in the context of the sharing economy, we solve the theoretical tensions in the distinctiveness-performance relationship by demonstrating configurational equifinality—both radical and conservative strategies succeed when external and internal dimensions achieve strategic coherence. Third, advancing research of sharing user participation, we establish that participation in trust-sensitive sharing platforms depends on strategic alignment between positioning and governance dimensions rather than isolated optimization of individual dimensions. These contextualized findings provide managers with evidence-based frameworks to coordinate market positioning strategies with governance investments, particularly when operating in institutionally complex environments. The demonstrated mechanisms in China’s sharing economy warrant future verification in other platform types and cultural contexts.

## Introduction

In the digital economy’s competitive landscape, platform firms confront a strategic paradox: reconciling differentiation-driven competitive advantage [1] with conformity-based institutional legitimacy [2–4]. Strategic management scholars emphasize how firms must distinguish themselves from competitors to achieve competitive advantage [1]. Doing so requires managers to deviate from typical approaches and develop new positions that competitors may have difficulty understanding [2]. At the same time, institutional theorists stress the importance of positioning similar to other firms, arguing that conformity allows organizations to gain legitimacy and avoid penalties for deviant behavior [3,4]. This tension manifests acutely in the sharing economy, where pioneers like Airbnb and Didi exemplify how innovative positioning disrupts traditional industries, yet governance failures perpetuate systemic trust crises [5].

While scholarly consensus advocates strategic equilibrium between legitimacy acquisition and competitive differentiation [6–8], the operationalization of optimal distinctiveness theory [9] remains constrained by two critical limitations. First, scholars conflate strategic differentiation with narrow product-market positioning (e.g., [10,11]), thereby neglecting the multidimensional nature of competitive strategies that simultaneously span product offerings, user segmentation, and geographic specialization [1]. Second, the predominant assumption that platform ecosystems achieve competitiveness through singular dimensions fails to account for governance behaviors’ moderating role in institutional legitimacy [12]. These dual reductions culminate in a theoretical impasse: While optimal distinctiveness theory prescribes balancing conformity and differentiation [9], it fails to explain how platforms reconcile aggressive positioning with institutional legitimacy when governance systems are excluded from strategic calculus. Consequently, the literature oversights engender persistent strategic dilemmas—radical innovation risks legitimacy erosion, while conservative imitation fosters competitive homogenization.

This impasse is magnified in the sharing economy’s transactional trilemma, where three endemic challenges intersect: (1) anonymity in peer interactions, which erodes trust due to the absence of institutional safeguards [5]; (2) pre-transaction uncertainties (e.g., landlord may conceal the true condition of the shared house), which heightens exposure for both providers and users [13]; and (3) regulatory ambiguity, which leaves platforms legally contested over liability standards [14]. Under these conditions, the governance system structure affects the legitimacy of the platform and becomes a key factor for user participation [14]. For example, Didi’s aggressive differentiation through dynamic pricing initially captured market share but triggered legitimacy crises when governance lapses (e.g., inadequate driver screening) resulted in safety scandals. Conversely, overly conservative platforms that prioritize institutional compliance often stagnate through service homogenization (e.g., generic home-sharing protocols mimicking hotel standards). Thus, the sharing economy’s unique dynamics demand the strategies that synchronize external market positioning with adaptive governance architectures, rather than optimizing either dimension in isolation.

To address these theoretical and empirical gaps, we integrate Porterian positioning theory [1] with platform boundary theory [15,16], developing an analytical framework that operationalizes three external positioning dimensions (variety-based, gender-based, and region-based differentiation) alongside two internal governance dimensions (resource ownership and transaction process differentiation). This integration combines the internal and external perspectives of the platform, highlighting the importance of the synergy of internal and external strategies. Employing fuzzy-set Qualitative Comparative Analysis (fsQCA), we transcend traditional regression methods’ limitations by modeling nonlinear interdependencies among these dimensions [17]. Our analysis identifies two equifinal high-performance archetypes: an *aggressive differentiation strategy* characterized by market pioneering coupled with governance prudence, and a *conservative differentiation strategy* emphasizing market alignment with institutional compliance.

Three pivotal contributions emerge. First, we expand differentiation theory beyond product-centric views by incorporating governance dimensions [12], revealing how platforms navigate the legitimacy-differentiation paradox through compensatory alignment. Second, we resolve theoretical tensions in the distinctiveness-performance relationship [11] by demonstrating configurational equifinality—both radical and conservative strategies succeed when external and internal dimensions achieve strategic coherence. Third, we establish that sharing platforms’ success derives not from singular optimal positioning but from systemic orchestration of the differentiation of market positioning and governance architectures-a configuration particularly critical in trust-sensitive environments where user participation hinges on perceived transactional security [18].

## Literature review

### Conceptual foundations

#### Platform ecosystems.

The theoretical construct of platform ecosystems emerged through the synthesis of modular systems theory and two-sided market principles [19,20]. Distinct from biological ecosystem analogies, platform ecosystems are characterized by purpose-driven coordination mechanisms that align participants around a central technological architecture. Building on Adner’s [21] seminal work, we conceptualize platform ecosystems as *value co-creation networks* where platform owners, complementors (supply-side users), and consumers (demand-side users) collectively fulfill shared value propositions through structured interactions. This tripartite structure comprises the core platform infrastructure, multi-homing participant groups (suppliers/consumers) and governance entities managing ecosystem rules.

Value generation in these ecosystems fundamentally stems from network effect dynamics. Following Eisenmann et al.‘s [22] market definition framework, platform markets exhibit dual network effects: same-side network effects and cross-side network effects. Same-side network effects refers to the user utility increases with participant density within market sides [23]. And, cross-side network effects refers to that value accretion on one market side depends on participant characteristics and scale on the opposing side [24]. These effects create self-reinforcing cycles that enable dominant platforms to capture disproportionate market shares through “virtuous cycle” dynamics [25]. However, such dominance necessitates sophisticated governance mechanisms to balance value creation and appropriation [13].

**Fig 1:**
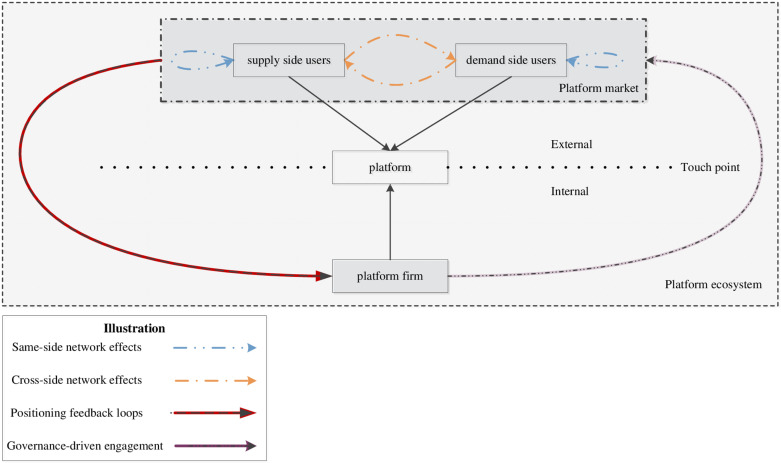
Ecosystem structure and interaction of sharing platforms.

Platform ecosystems demonstrate recursive relationships between governance strategies and market positioning. As depicted in Fig 1, this interdependence manifests through two key mechanisms. One is *governance-driven engagement*. Platform firms orchestrate participation through rule systems that incentivize desired behaviors while mitigating opportunism [12,13]. The other one is *positioning feedback loops*. Market positioning influences governance design by altering competitive pressures and stakeholder power dynamics [6,11]. Governance rules must co-evolve with positioning strategies to maintain ecosystem coherence [13,26].

#### Platform.

Despite the ubiquity of “platform” terminology in management research, conceptual fragmentation persists across disciplinary lenses [27]. Our synthesis integrates technological and economic perspectives, viewing the platform as a technological architecture that coordinates multilateral markets and provides the necessary infrastructure for open innovation [28].

The techno-economic paradigm reveals platforms as institutionalized innovation arenas that perform three synergistic functions: First, they coordinate multilateral transactions through algorithmic matching systems that reduce search frictions and optimize resource allocation. Second, by establishing standardized infrastructure for value exchange, platforms create scalable ecosystems where heterogeneous participants can transact under unified protocols. Third, through API-mediated complementarity [28], they enable combinatorial innovation where third-party developers can build upon core functionalities to create novel services. This techno-economic duality – simultaneously operating as technical architectures and market intermediaries – generates critical strategic imperatives for sharing platforms. Their technical systems must be architected to reconcile two seemingly contradictory requirements: maintaining sufficient standardization to ensure ecosystem interoperability while allowing customizable differentiation at the service layer to capture niche market opportunities. Thus, it serves as a point of interaction for internal platform enterprises and external platform markets.

#### The sharing economy context.

Recent empirical work reveals that effective platforms achieve *dynamic complementarity*– synchronizing governance adjustments with positioning shifts to exploit emerging market opportunities [11]. This theoretical lens proves particularly salient when examining sharing economy contexts.

The sharing economy’s rapid ascension as a disruptive economic paradigm has generated intense scholarly debate regarding its conceptual boundaries [29–31]. Despite the lack of a unified definition, these various interpretations converge on the central role of sharing platforms in mediating transactions with external parties [32]. This role highlights the strategic placement of sharing platforms within the platform ecosystem, where they are instrumental in resource allocation among a spectrum of stakeholders, governed by distinct architectural frameworks. Accordingly, this paper frames the sharing economy as a platform-centric service economy that facilitates the convergence of supply and demand by orchestrating the utilization of underutilized resources. This conceptualization not only encapsulates the fundamental nature of the sharing economy but also resonates with the operational mandate of platforms within this ecosystem

A platform that coordinates the sharing economy market and provides its infrastructure is defined as a sharing platform, while the enterprise responsible for managing the platform is referred to as the platform enterprise [33]. Table 1 summarizes the related terms and definitions discussed in this paper.

**Table 1:** Related conceptions and definitions.

Conception	Definition	Key References
Sharing economy	A service economy based on platform coordination of idle resources to match supply and demand	[29,30,34]
Sharing platform	A technical system that coordinates and provides infrastructure for sharing economy markets	[35,36]
Platform company	The leading company responsible for the sharing platform	[33]
Platform market	The market of users served by the sharing platform through its functions	[22]
Platform governance	The strategies developed and implemented by platform companies to create and appropriate value	[13]
Platform ecosystem	An alliance structure formed by a group of platform-centered members for the purpose of a common value proposition	[21,37]

### Platform differentiation and performance nexus

The academic discourse surrounding platform differentiation reveals a persistent theoretical impasse rooted in two conflicting perspectives. The initial research stream focuses on strategic differentiation mechanisms, investigating competitive positioning strategies through product/service innovation via feature differentiation [38,39] and market segmentation via selective user targeting [40]. Recent extensions of this work explore governance-based differentiation, particularly examining how information privacy management systems can serve as competitive demarcation tools [12]. Concurrently, a parallel investigates optimal distinctiveness thresholds, yielding contradictory empirical patterns: while some studies posit an inverted U-curve relationship where moderate distinctiveness maximizes performance by balancing conformity and innovation [7,9], others demonstrate U-shaped outcomes where extreme strategies—either full differentiation or complete conformity—consistently outperform intermediate approaches [6]. These contradictions are further complicated by evidence of context-dependent effects where environmental contingencies reverse strategic directionality [8,10,41,42], collectively challenging the foundational assumptions of the strategic balance paradigm [42–44].

This theoretical ambiguity stems from co-occurring conceptual and methodological limitations. Conceptually, the prevailing conceptual framework suffers from reductionist oversimplification, disproportionately emphasizing product-market [6] or user-segment differentiation [7], neglecting the ecosystemic interdependencies inherent to platform competition [33]. Platform differentiation is not merely a unilateral positioning choice but a dynamic interplay of cross-side network effects (e.g., how supply-side scale amplifies demand-side value), complementor co-innovation (e.g., third-party developers extending core functionalities), and governance architecture (e.g., reinforce privacy protocols) [45]. Methodologically, traditional variable-centered approaches—particularly their focus on linear net effects [41]—obscure the causal complexity underlying differentiation-performance relationships. By isolating individual dimensions, these methods fail to capture the synergy of multiple factors, equifinal pathways to success (e.g., aggressive vs. conservative strategies achieving similar outcomes through distinct governance-positioning alignments) [42]. This dual limitation—conceptual narrowness reinforcing methodological inadequacy—perpetuates a self-reinforcing cycle of contradictory evidence and theoretical stagnation.

Our study addresses these theoretical and methodological gaps through two pivotal innovations that collectively resolve the differentiation-conformity stalemate. First, we transcend reductionist conceptions of differentiation by integrating Porterian positioning theory [1] with platform boundary theory [15,16], thereby redefining differentiation as a multidimensional construct that spans both external market positioning and internal governance architectures. While Porterian theory illuminates how firms achieve competitive advantage through external strategies like product differentiation, user segmentation, and geographic specialization, it remains silent on how platforms internally govern the legitimacy risks such differentiation. Conversely, platform boundary theory explicates governance mechanisms for ecosystem coordination, but lacks analytical tools to connect these mechanisms with market positioning strategies. This synthesis reveals that sustainable differentiation emerges not from trading off market innovation against institutional conformity, but from strategically aligning external and internal dimensions to achieve synergistic effect.

Second, we employ fuzzy-set Qualitative Comparative Analysis (fsQCA) to model the nonlinear interdependencies between these dimensions, moving beyond the constraints of traditional regression methods that isolate linear net effects. Where conventional approaches obscure causal complexity by analyzing variables in isolation, fsQCA captures how specific combinations of external positioning and internal governance generate competitive advantage. This methodological shift uncovers equifinal pathways to success.

## Theoretical framework

**Fig 2:**
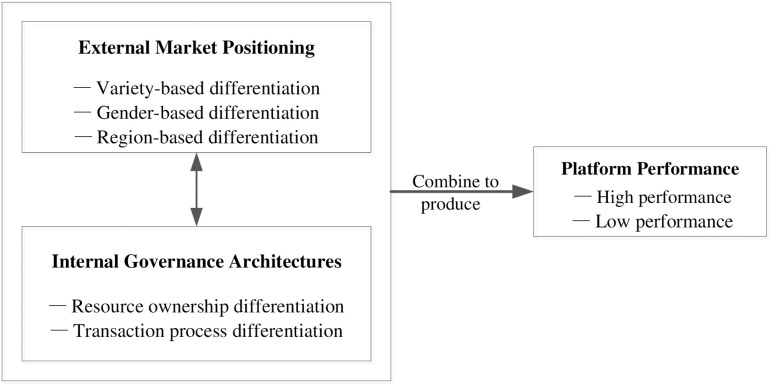
Theoretical framework.

Grounded in the premise that platform viability depends on synergistic alignment between external market positioning and internal governance architectures, our model (Fig 2) conceptualizes distinctiveness as a dual-dimensional construct requiring simultaneous optimization of ecosystem boundaries and value propositions.

### External market positioning

Building on Porter’s [1] strategic positioning paradigm, we operationalize external distinctiveness through three interdependent dimensions.

#### Variety-based differentiation.

Variety-based differentiation positioning prioritizes product/service portfolio differentiation over customer segmentation, employing Nelson’s [46] product typology framework that classifies offerings as search goods (pre-purchase evaluable attributes), experience goods (post-consumption quality assessment), or credence goods (persistent evaluation challenges). In sharing economy contexts, this categorization becomes particularly salient given the heightened behavioral uncertainty stemming from underutilized asset utilization [31,47]. While strategic conformity with market leaders enhances institutional legitimacy [10], excessive alignment risks commoditization, necessitating calibrated differentiation to balance legitimacy and competitive advantage.

#### Gender-based differentiation.

Gender-based differentiation positioning introduces unique complexities in peer-to-peer exchange environments. Unlike traditional markets, sharing transactions frequently occur in private domains (residences, personal vehicles), amplifying user preferences for physical security and emotional comfort [48]. This intimacy imperative creates gendered market dynamics where female participants demonstrate higher trust capital [49] and superior economic returns [50], incentivizing platforms to develop gender-specific positioning strategies. However, such specialization involves strategic trade-offs – while circumventing direct competition with dominant players [6], it confines platforms to narrower market niches with constrained profitability potential.

#### Region-based differentiation.

Complementing geographic considerations, region-based positioning recognizes the spatial embeddedness of platform ecosystems. Ride-hailing and homestay platforms exemplify how network effects remain territorially bounded [51], with regional cultural norms and regulatory regimes critically mediating scalability [52]. The failure of Airbnb’s standardized global model in China underscores the imperative for localized adaptation, where insufficient attention to regional distinctiveness can precipitate market exit despite global dominance (Airbnb places third in a ranking of the leading online travel agencies by revenue and the third most visited travel and tourism website worldwide in 2025) (Data from: https://www.statista.com/topics/2273/airbnb/#topicOverview). Geographic positioning thus represents both opportunity and constraint – enabling focused resource deployment while necessitating sustained investment in local ecosystem cultivation [53].

### Internal governance architectures

Platform competitiveness equally hinges on governance mechanisms that balance ecosystem openness with quality control [13]. Platform governance begins with delineating the platform’s boundaries, that is, identifying the assets it controls and the activities it undertakes [15,16]. This enables the platform firm to determine, through incentive and control mechanisms, which value-creating activities to foster on the platform [13,26]. Consequently, drawing from boundary theory [15,16], we analyze governance distinctiveness through resource ownership models and transaction process design.

#### Resource ownership differentiation.

This metric measures the ownership of shared products by the focal sharing platform [54]. Asset ownership strategies span a continuum from capital-intensive models (e.g., Zipcar’s proprietary fleets) to asset-light approaches leveraging user-owned resources (e.g., Lyft’s peer-sourced vehicles) [55]. While lean ownership structures reduce operational costs [31], they introduce quality assurance challenges that demand sophisticated reputation systems – a paradox where cost efficiency competes with service reliability.

#### Transaction process differentiation.

Transaction process design governs value co-creation through rights allocation between platform operators and users. Strategic empowerment of participants through transparent review systems and flexible booking protocols enhances transaction efficiency [56,57], yet excessive decentralization risks ecosystem fragmentation. Unchecked user autonomy correlates with quality deterioration [58,59] and operational complexity escalation [13], necessitating governance architectures that intermediate between participant freedom and platform oversight. This delicate balance becomes particularly crucial in sharing contexts where trust mechanisms must offset the inherent risks of stranger-mediated exchanges.

## Data and methodology

### Data collection

Given mobile applications’ critical role in sharing platform success [60], our study analyzes operational data from Chinese sharing platforms with active mobile apps. The sample construction process employed three exclusion criteria to ensure conceptual alignment and data reliability. Platforms were excluded if they (1) originated from traditional market expansions to avoid legacy performance distortions, (2) operated in financial sharing sectors with unique transaction logics, or (3) lacked verified active user metrics and demographic data from TalkingData. After implementing these filters and removing incomplete entries, our final sample comprised 33 sharing platforms across five market segments, listing in S2 Table.

We triangulated data from three complementary sources to operationalize key constructs: First, platform performance metrics and demographic positioning data (gender/region) were sourced from TalkingData’s mobile analytics platform, covering 770 million active devices. Second, product variety positioning indicators were extracted from Qimai Data’s global app store intelligence system, spanning 155 countries and nine Android markets. Third, governance behavior variables (resource ownership and transaction processes) were codified from platform rulebooks, terms of service documents, and official corporate disclosures.

### Variable measurement

#### Outcome variable.

Platform performance was operationalized as the ratio of a focal platform’s monthly active users (MAU) to the industry leader’s MAU in the preceding month, standardized by market segment. This relative performance metric captures competitive dynamics in mature markets where user retention determines long-term viability [61]. The leader-platform benchmarking approach, consistent with Barlow [10], controls for pre-existing market structures while enabling cross-platform comparability. This normalization logic extends to our distinctiveness measurements, where deviation from industry norms quantifies strategic positioning choices.

#### Condition variables.

Adopting Barlow et al.’s [10] benchmarking framework, we quantify platform distinctiveness through systematic deviation from sector leaders – defined as platforms with maximal monthly user engagement in their respective domains. This leader-referenced approach captures the “winner-takes-most” dynamics inherent to platform economies while controlling for pre-existing market structures.

***External market positioning***. *Variety-based differentiation* is measured as the categorical divergence in product/service offerings, calculated through the absolute difference in composite scores between focal and leader platforms. Following Nelson’s [46]typology, each platform’s product portfolio is scored as the sum of binary indicators (1 = present, 0 = absent) for search goods, experience goods, and credence goods, derived from application description texts mandated for app store submissions [10]. Higher scores reflect greater differentiation from market norms, with maximal divergence occurring when platforms specialize in distinct good types. *Gender-based differentiation* captures demographic targeting differences through the absolute discrepancy in female user ratios $abs(y_{i}-y)$, where $y_{i}$ and y represent focal and leader platform proportions respectively. This metric recognizes the strategic importance of gender dynamics in sharing economies, where female participants demonstrate higher trust capital and transaction frequency. *Region-based differentiation* extends this logic to geographic markets, computed as the summed absolute differences in user distribution across seven Chinese regions (East, South, North, Central, Southwest, Northwest, Northeast). The regional classification accounts for cultural, economic, and regulatory heterogeneity influencing platform adoption patterns.

***Internal governance architectures.*** *Resource ownership differentiation* evaluates asset strategy divergence using a three-tier ordinal scale: full ownership (3), partial ownership (2), or complete outsourcing (1). The absolute difference $abs(m_{i}-m)$ between focal and leader platform scores reflects strategic positioning along the asset-light continuum, balancing cost efficiency against quality control imperatives. Using Didi as the benchmark platform, Dida maintains strategic alignment by adopting an identical asset-light operational model. Consequently, Dida’s resource ownership differentiation score registers 0.

*Transaction process differentiation* adopts a granular activity-based analysis [18], comparing eight sequential transaction phases from pre-transaction promotion to post-transaction management (e.g., user source, product or service review, demand release, matching transactions, transaction execution, risk control, conflict resolutions and service quality management). Each phase deviation scores 1 (divergent) or 0 (convergent), with the composite index ($(n_{1}+\ldots +n_{i}+\ldots n_{8})/8$)reflecting overall process innovation. Using Didi as the benchmark platform, Dida maintains strategic alignment in five operational dimensions: user source (e.g., ground promotion), product or service review (e.g., driver qualification audits), risk control (e.g., itinerary sharing and emergency alert features), conflict resolution mechanisms (e.g., online customer service), and service quality management (e.g., rating systems and post-service surveys). However, it implements differentiation in three dimensions: demand release (enabling both drivers and passengers to post itineraries, unlike Didi’s passenger-only posting), transaction matching (omitting system dispatching orders present in Didi’s system), and transaction execution (permitting driver-passenger route negotiations versus Didi’s mandatory navigation compliance). This results in a transaction process differentiation score of 3/8 across eight core functional dimensions.

To assess the consistency of each transaction activity on the focal platform with that of the leading platform, this paper employs a two-pronged methodology: (1) quantifying variables through an examination of the regulations published by each sharing platform, and (2) validating the preliminary quantifications by participating in transactions on these platforms using demand-side user accounts. Definitions of these variables are provided in Table 2.

**Table 2:** Variable Definitions.

Variables	Sample Size
**Outcome variable**	
Platform performance	The ratio of monthly active users between focal and leader platforms
**Condition variables**	
Variety-based differentiation	The absolute gap in product/service offerings between focal and leader platforms
Gender-based differentiation	The absolute gap in female user proportions between focal and leader platforms
Region-based differentiation	The absolute gap in regions between focal and leader platforms
Resource ownership differentiation	The absolute gap in asset strategy between focal and leader platforms
Transaction process differentiation	The absolute gap in transaction phases between focal and leader platforms

Tables 3 and 4 present descriptive statistics and correlation matrices, respectively, establishing baseline characteristics and mitigating multicollinearity concerns.

**Table 3:** Descriptive statistics.

Variables	Sample Size	Mean	St.dev	Min	Max
Platform performance	33	0.3230	0.3709	0.0025	1.1474
Variety-based differentiation	33	0.3636	0.5488	0.0000	2.0000
Gender-based differentiation	33	0.1035	0.1046	0.0007	0.4156
Region-based differentiation	33	0.1394	0.0869	0.0203	0.3908
Resource ownership differentiation	33	0.8485	0.9395	0.0000	2.0000
Transaction process differentiation	33	0.3409	0.2782	0.0000	0.7500

**Table 4:** Correlation analysis.

Variables	1	2	3	4	5	6
1. Platform performance	1.0000					
2. Variety-based differentiation	−0.0114	1.0000				
3. Gender-based differentiation	0.0247	−0.2403	1.0000			
4. Region-based differentiation	0. 0072	−0.3252^*^	0.0009	1.0000		
5. Resource ownership differentiation	−0.4365^**^	0.3527^**^	−0.1294	−0.2288	1.0000	
6. Transaction process differentiation	−0.6007^***^	0.3140^*^	−0.2296	−0.3107^*^	0.6671^***^	1.000

### Research method

Employing fsQCA through specialized computational software (fsQCA3.0), we examine the configurational complexity underlying platform performance [62]. Rooted in set-theoretic principles and Boolean algebra, fsQCA enables systematic identification of causal condition combinations sufficient for outcome attainment [63]. This methodology operationalizes variables on a continuous [0,1] scale, where 0 signifies absolute non-membership and 1 represents full membership in target sets, thereby accommodating partial membership gradations essential for capturing strategic nuance.

The selection of fsQCA over conventional regression techniques stems from its unique capacity to address three critical challenges in platform strategy research. First, our analytical framework involves complex patterns of causal condition combinations that generate either over-differentiated positioning (high differentiation) or non-differentiated positioning (low differentiation). FsQCA is uniquely suited to examine such complexity, as it accommodates causal asymmetry between outcomes. Furthermore, given the abductive nature of this research, we lacked explicit a priori expectations about causal condition combinations. In standard regression analysis, modeling all possible interactions to cover every contingency would likely yield computationally infeasible specifications, with higher-order interaction coefficients proving particularly difficult to interpret [17,64,65].

Second, multiple distinct solutions may exist for the same outcome—a phenomenon termed equifinality that is challenging to model in regression analysis. For instance, one solution for a given outcome might require the presence of a specific antecedent condition, while a second solution for the same outcome could necessitate the absence of that identical condition [62]. In regression analysis, such countervailing effects might neutralize each other, potentially rendering the antecedent condition statistically insignificant [65]. In contrast, fsQCA is explicitly designed to detect and permit equifinal solutions, thereby enabling analysis of complex asymmetric relationships among antecedent conditions.

Finally, fsQCA’s conclusions are less susceptible to distortion from outliers [17,66] or endogeneity arising from omitted variable bias [67,68], as the method makes no assumptions about underlying distributions and does not rely on correlational analysis. Within fsQCA, outliers either fail to meet the minimum consistency thresholds required for solution inclusion or are automatically mitigated through low coverage parameters that researchers can define to limit explanatory scope. Additionally, since fsQCA does not estimate coefficients for individual explanatory factors, it avoids the partial effect biases inherent in regression models when existing variables capture portions of omitted variable effects [62].

### Calibration

Our study requires the calibration of variables into fuzzy sets of causal conditions and outcomes, spanning from 0 (absolute non-membership) to 1 (full membership) [17]. To ensure methodological alignment, this includes adherence to a calibration threshold for continuous variables [69]: adopting the 95% quartile for full membership, the 50% quartile as the crossover point, and the 5% quartile indicating full non-membership. For discrete variables, it is calibrated by the direct method [67,70]. Ultimately, based on the sample consistency data characteristics, combinations with at least one empirical instance and minimum consistencies of 0.8 (raw) and 0.75 (PRI) are reliably identified as delivering high and low platform performances. Calibration results are provided in Table 5.

**Table 5:** Calibration results.

Variables	Full membership	Cross-over	Full non-membership
Platform performance	1.04	0.12	0.01
Variety-based differentiation	2	1	0
Gender-based differentiation	0.31	0.07	0.004
Region-based differentiation	0.32	0.11	0.05
Resource ownership differentiation	2	1	0
Transaction process differentiation	0.75	0.25	0

## Results

### Necessary conditions analysis

Prior to configuration exploration, we established the absence of individually necessary conditions through formal necessity testing. Following QCA methodological protocols [67], each condition variable (including negated forms) was assessed for its indispensability in achieving high/low platform performance. The consistency thresholds, which quantify the degree to which a condition is consistently present when the outcome occurs, remained below the critical 0.9 benchmark across all variables (Table 6). This empirical evidence conclusively demonstrates that no single strategic dimension – whether in market positioning or governance design – constitutes a standalone prerequisite for performance outcomes, necessitating configurational analysis to uncover complex interdependencies.

**Table 6:** Necessary Conditions Analysis.

Conditional Variables	High performance		Low performance	
	Consistency	Coverage	Consistency	Coverage
High variety-based differentiation	0.32	0.71	0.29	0.72
Low variety-based differentiation	0.87	0.53	0.88	0.58
High gender-based differentiation	0.64	0.68	0.50	0.58
Low gender-based differentiation	0.61	0.53	0.73	0.69
High region-based differentiation	0.60	0.61	0.58	0.63
Low region-based differentiation	0.63	0.58	0.64	0.64
High resource ownership differentiation	0.37	0.41	0.64	0.77
Low resource ownership differentiation	0.79	0.67	0.51	0.47
High transaction process differentiation	0.52	0.47	0.80	0.79
Low transaction process differentiation	0.76	0.78	0.45	0.51

### Truth table analysis

Truth table analysis revealed two equifinal pathways to high performance (H1-H2) and two distinct failure trajectories (L1-L2), as detailed in Table 7. The high-performance configurations exhibit strategic complementarity between governance standardization and market specialization: Configuration H1 combines low governance distinctiveness with concentrated regional positioning, suggesting efficiency advantages in mature markets. Conversely, Configuration H2 achieves superiority through radical market differentiation paired with adaptive governance, indicating disruptive innovation potential. The low-performance archetypes emerge from strategic misalignment – Configuration L1’s moderate market differentiation proves insufficient to offset the costs of governance complexity, while Configuration L2’s governance over-differentiation erodes the benefits of market conformity. These patterns fundamentally challenge linear conceptions of strategic distinctiveness, demonstrating that both hyper-specialization and balanced ambidexterity can succeed contingent on coherent configuration design.

**Table 7:** Configurations of high and low platform performance.

Conditional Variables	High performance		Low performance	
	H1	H2	L1	L2
**External market positioning**				
Variety-based differentiation	●		⊗	
Gender-based differentiation	●	⨂		⊗
Region-based differentiation	●	⊗	●	
**Internal governance architectures**				
Resource ownership differentiation	⨂	⨂		●
Transaction process differentiation		⊗	●	•
Consistency	0.89	0.99	0.89	0.94
Original Coverage	0.3	0.17	0.3	0.5
Unique Coverage	0.16	0.03	0.16	0.25
Overall Consistency	0.90		0.92	
Overall Coverage	0.34		0.70	

**Notes**: Large circles indicate core elements and small circles denote peripheral elements. A filled circle (●) signifies the presence of a condition and an empty circle with a cross (⊗) denotes its absence.

### Validation and robustness tests

Referring to Fiss [17], this study verifies the acceptability of our solutions with the measurement of two metrics: consistency and coverage. Generally, solution consistency levels should exceed 0.75 [17,67,68], while coverage reflects the empirical relevance or importance of configurations [67], analogous to R2 in regression analysis [17], with no fixed threshold for this metric. The overall consistency of our high and low platform performance are 0.90 and 0.92, surpassing the traditionally accepted threshold of 0.8 [17]. Additionally, the observed coverage values for high and low platform performance are 0.34 and 0.70, aligning with suggested acceptable ranges, such as the coverage threshold of 0.27 proposed in prior work [17].

To validate research findings, we apply an assortment of verification methods. First, we adjusted the consistency threshold from 0.8 to 0.85 while maintaining the original frequency threshold and PRI consistency levels. This modification yielded unchanged overall solution consistency and coverage measures across performance configurations, with all causal recipes being fully reproduced.

Second, we relaxed the PRI consistency threshold from 0.75 to 0.7 while preserving the frequency and primary consistency thresholds. This adjustment resulted in marginal variations: the overall consistency for high-performance configurations decreased to 0.86 while their coverage increased to 0.45. For low-performance configurations, the overall consistency remained stable, yet coverage improved to 0.72. All causal recipes were again fully reproduced.

Third, we modified the calibration criteria by replacing the 95%/50%/5% calibration cutoffs with 85%/50%/15% thresholds while retaining the frequency threshold of 1, consistency threshold of 0.8, and PRI consistency threshold of 0.75. Subsequent analysis demonstrated no changes in overall consistency or coverage measures for either performance configuration, with complete replication of all causal recipes.

This multi-method validation approach mitigates overfitting concerns while affirming the configurations’ theoretical generalizability beyond sample-specific characteristics. Moreover, the solution set’s validity is substantiated through dual lenses of statistical rigor and parametric sensitivity.

## Theoretical propositions

Building on optimal distinctiveness theory, our configurational analysis reveals two equifinal strategic archetypes driving platform success in sharing economies: the *aggressive differentiation strategy* prioritizing market novelty, and the *conservative differentiation strategy* emphasizing governance alignment. The interplay between these models elucidates how platforms navigate the legitimacy-differentiation paradox through distinct ecosystem orchestration strategies.

### Aggressive differentiation strategy

Configurations H1 and L1 collectively delineate an aggressive differentiation paradigm where radical market positioning combines with restrained governance innovation. This strategic archetype emerges when platforms operate under conditions of fragmented consumer preferences [24,71] and low multi-homing costs, enabling coexistence through niche specialization [45]. By targeting underserved market segments – whether through gender-specific user targeting, regional concentration, or novel product categorization – platforms circumvent direct competition with incumbents while capitalizing on first-mover advantages in emerging niches [6].

The case of Mayi epitomizes this strategic calculus. Facing Airbnb’s dominance in China’s accommodation-sharing market, Mayi adopted tripartite differentiation: 1) exclusive focus on house-sharing (versus Airbnb’s experiential offerings), 2) male-dominated user targeting (58% vs. 44%), and 3) concentrated operations in Southwest China’s secondary cities where incumbent penetration remained limited. This market novelty strategy, however, necessitated counterbalancing governance conformity to mitigate the trust deficit inherent in niche markets. By systematically replicating trust-building mechanisms from industry leaders Airbnb—such as two-way identity verification (e.g., mandatory real-name registration for both hosts and guests) and credit system integration (e.g., incorporating third-party credit systems like Alipay’s Sesame Credit)—Mayi offset the legitimacy risks of market deviance while preserving differentiation benefits. The strategic equilibrium demonstrates how aggressive distinctiveness succeeds not through unilateral differentiation, but via compensatory alignment between market novelty and governance familiarity.

This model’s inherent risks surface in Configuration L1, where moderate governance distinctiveness combined with market novelty erodes performance. The deterioration highlights the criticality of maintaining governance alignment thresholds – excessive rule customization in trust-sensitive sharing contexts undermines transactional reliability, negating market differentiation advantages [5,13]. Fengniao serves as a counterexample. Compared to industry leader Ishansong, Fengniao strategically targets male-dominated demographics and southern markets to avoid direct rivalry. However, it diverged significantly from Ishansong’s governance architectures by adopting an asset-heavy operational model and implementing an order-grabbing mechanism for riders. These structural deviations induced unintended consequences: the order-grabbing system created perceived inequities as some riders persistently secured no orders, while the asset-intensive approach escalated operational expenditures. Collectively, these governance choices contributed to Fengniao’s suboptimal performance. We therefore posit:

**Proposition 1**: Aggressive differentiation strategy yields superior performance when platforms couple radical market positioning with governance architectures that alignment with institutional norms, creating compensatory legitimacy to offset differentiation risks.

### Conservative differentiation strategy

Configurations H2 and L2 unveil a conservative strategic paradigm were platform success stems from synchronized alignment with market leaders’ positioning and governance norms. This approach emerges under conditions of entrenched network effects and high consumer switching costs, where differentiation risks triggering “winner-takes-more” dynamics [6]. In such environments, challenger platforms confront dual pressures: intense competition for mass-market users and legitimacy deficits when deviating from institutionalized practices.

The strategic rationale for conservative differentiation operates through two reinforcing mechanisms. First, market positioning conformity capitalizes on the “halo effect” – users inherently associate leader platforms’ market dominance with service quality and reliability [10]. By mirroring leader geographies and demographics, challenger platforms piggyback on established user perceptions while minimizing cognitive switching costs. Second, governance alignment addresses the trust paradox in sharing economies: users perceive leader-designed transaction protocols as institutional safeguards against privacy breaches and opportunistic behavior [5]. Radical governance innovation, conversely, amplifies risk perceptions by disrupting users’ established mental models of transactional security.

Tujia’s strategic trajectory exemplifies this dual-alignment logic. While competing directly with Airbnb in China’s mainstream accommodation market, Tujia maintains near-identical user demographics (50.4% female vs. Airbnb’s 56%) and geographic overlap (Δ = 0.09). This situation subjects Tujia to intensified market competition. To avoid falling into the dual dilemma of strong competitive pressure and heightened legitimacy demands, Tujia meticulously replicates Airbnb’s governance architecture with zero behavioral distinctiveness. This governance isomorphism capitalizes on Airbnb’s “halo effect”, reinforcing user recognition of Tujia while minimizing adaptation costs. Such strategic demonstrates how conservative differentiation converts institutional constraints into competitive advantages through systematic emulation of market-leading platforms.

The configuration’s fragility surfaces in L2, where marginal market differentiation combines with governance novelty, depressing performance compared to H2. This divergence underscores the primacy of governance conformity in trust-sensitive contexts – even minor rule modifications can trigger disproportionate risk perceptions when market positioning lacks compensatory differentiation. Huochebang serves as a counterexample. By directly competing with industry leader Huolala in the mass market (given their similar market positioning structures), Huochebang not only faced intense competitive pressure but also encountered consumer perceptions shaped by Huolala’s dominance, which solidified fixed expectations regarding industry governance architectures. Despite this, Huochebang attempted governance innovations, such as adopting a bid-based order allocation system instead of Huolala’s fixed-price order-grabbing model, and resolving disputes through mediation rather than Huolala’s approach of determining fault within 12 hours while withholding driver deposits. These novel governance practices, however, lacked consumer acceptance, subjecting Huochebang to dual pressures of competitive disadvantage and legitimacy deficit, ultimately leading to its suboptimal performance. We therefore advance:

Proposition 2: Conservative differentiation strategy achieves superior performance when platforms deviation from leader platforms’ market positioning and replicate their governance protocols, leveraging institutional legitimacy to offset late-mover disadvantages.

## Conclusions

This investigation elucidates the strategic symbiosis between governance architectures and market positioning in shaping sharing platform competitiveness. Through an ecosystem lens, we demonstrate that platform differentiation transcends unilateral strategic choices, requiring alignment of external value propositions with internal rule systems. The application of configurational analysis (fsQCA) reveals two equifinal pathways to superior performance—aggressive market pioneering with governance prudence, and conservative market alignment with institutional compliance—thereby redefining optimal distinctiveness as a dynamic equilibrium rather than static positioning.

### Theoretical implications

Our findings yield three pivotal advancements to platform strategy literature. First, by integrating Porterian positioning theory with platform boundary theory, we reframe differentiation through ecosystem interdependence, establishing a novel theoretical lens for analyzing differentiation strategies. This integration achieves dual breakthroughs: (1) resolving the persistent product-governance dichotomy in platform literature [6,12] through extending Porter’s theory to multi-sided markets, and (2) demonstrating through fsQCA analysis of Chinese sharing platforms that competitive advantage emerges not from isolated “positioning or governance” choices [7], but from their architectural alignment–a systemic perspective absent in current platform boundary theories [33]. Specifically, governance structures first enable novel positioning strategies, which subsequently necessitate governance adaptation, thereby redefining differentiation as an emergent outcome of ecosystem orchestration. These findings challenge reductionist models by establishing a contingency framework for alignment patterns in sharing platforms, while urging future research to extend alignment analysis to other platform archetypes using the fsQCA methodology developed here.

Second, we advance optimal distinctiveness theory by demonstrating how institutional context shapes viable differentiation strategies in sharing economy platforms. Contrary to the presumed universal superiority of moderate differentiation [52] or context-free radical positioning [11], our configurational approach reveals that both extremes can succeed contingent on compensating mechanisms. The aggressive model counterbalances market novelty with governance conformity to mitigate legitimacy deficits, while the conservative approach offsets market mimicry through governance replication. This dual equilibrium model extends distinctiveness theory by demonstrating how institutional pressures [5] and network effect thresholds [6] jointly determine differentiation viability in the context of emerging economies.

Finally, our methodological application of fsQCA addresses the inherent complexity of alignment mechanisms between market positioning and governance architectures—a theoretical nexus central to understanding user participation in trust-sensitive sharing contexts. Traditional regression analyses, constrained by linearity assumptions [17], fail to capture the causal asymmetry where identical governance modifications produce divergent outcomes under varying market conditions. Our analysis of 33 Chinese sharing platforms demonstrates how fsQCA captures critical nonlinear dynamics that conventional approaches would miss. These findings provide methodological scaffolding for operationalizing the “strategic alignment” principle established in our user participation analysis, demonstrating how platform success in institutionally complex markets emerges not from isolated optimizations but from coherent positioning-governance architectures, which is particularly crucial in sharing economy contexts characterized by stranger-mediated transactions and asset specificity.

### Practical implications

The empirical insights from this study yield three actionable imperatives for sharing platforms navigating competitive ecosystems. First, platform owners should adopt a multidimensional differentiation strategy [72] that strategically coordinates market positioning with governance architectures, recognizing that high performance emerges from their systematic interdependence rather than isolated initiatives [73]. To operationalize this approach, managers should develop diagnostic frameworks such as the Strategic-Governance Alignment Matrix to empirically quantify interactions between market-oriented actions (e.g., niche targeting [74]) and institutional mechanisms (e.g., reputation systems [75]), thereby enabling resource prioritization toward dual-impact innovations that simultaneously enhance market distinctiveness and institutional credibility. Continuous monitoring of strategic alignment metrics should be institutionalized to rigorously assess the evolving synergies between market and institutional dimensions—a crucial practice given the liability of newness inherent in sharing economy ventures.

Second, the governance credibility imperative stemming from stranger-mediated transaction risks [76]. Our findings demonstrate that excessive governance distinctiveness erodes user trust by compared to industry-aligned practices, necessitating modular governance architectures that strategically integrate industry-aligned trust infrastructures with context-sensitive adaptations. Platform owners could prioritize standardized core modules—such as compulsory identity authentication protocols [77] and real-time transaction monitoring interfaces [78] mirroring sector leaders’ trust benchmarks—to establish baseline credibility. Simultaneously, they should implement localized functional modifications addressing empirically identified market needs, such as custom dispute resolution protocols for regions with institutional voids. This hybrid approach achieves optimal trust-innovation equilibrium, particularly crucial when targeting risk-averse user segments.

Lastly, this study demonstrates the viability of market segmentation strategies under constrained network effects [6]. By leveraging strategic niche specialization – as evidenced by Mayi’s successful Southwest China focus – late-mover platforms can secure market footholds against dominant incumbents through compensatory legitimacy strategies. This counterintuitive dynamic reveals opportunities in underserved geographic segments where localized network effects persist. Platform owners should therefore develop diagnostic frameworks like the Regional Network Effect Matrix [1], systematically assessing market-entry viability through two critical dimensions: segmentation-specific saturation thresholds (e.g., < 35% incumbent penetration) and institutional preparedness indicators (e.g., digital infrastructure maturity). Such tools enable data-driven identification of markets where compensatory legitimacy investments can optimally offset network effect disadvantages.

### Limitations and future research directions

While advancing platform strategy scholarship, this investigation acknowledges three boundary conditions requiring scholarly attention. The fsQCA outcomes highlight various routes to enhanced platform performance but overlook additional contextual elements. Further research is imperative to broaden the theory’s applicability by examining a range of contextual scenarios. The current sample, comprising 33 platform firms, is adequate for fsQCA but could be enlarged to uncover a more comprehensive array of distinctiveness strategies. Moreover, the findings’ scope is limited by the focus on the Chinese market, prompting a call for international data to enhance the robustness of future research conclusions. These limitations notwithstanding, the study establishes an empirical foundation for reimagining platform competition through ecosystem orchestration lenses, moving beyond reductionist single-variable paradigms toward holistic strategic frameworks.

## Supporting information

S1 Dataset Source data for statistical analyses. (XLSX)

S2 Table List of the platforms. (DOCX)
