# Supplementary material for: Strategic orchestration in sharing economies: A configurational analysis of platform differentiation and governance alignment
Source: PLoS One. 2025 Jun 25;20(6):e0326774. doi: 10.1371/journal.pone.0326774 (PMC12193835; doi:10.1371/journal.pone.0326774)
Supplement: S2 Table — (DOCX) [file pone.0326774.s002.docx]

**S2 Table. List of the platforms**

| **ID** | **Platform** |
| --- | --- |
| 1 | Aihuishou |
| 2 | Guazi |
| 3 | Renrenche |
| 4 | Goofish |
| 5 | Xin |
| 6 | Zhuanzhuan |
| 7 | Gofun |
| 8 | ofo |
| 9 | Caocao |
| 10 | Didi |
| 11 | Dida |
| 12 | Hello |
| 13 | Meituan |
| 14 | Mobike |
| 15 | Shenzhou |
| 16 | Zuche |
| 17 | 01zhuanche |
| 18 | 1haicn |
| 19 | Youonbike |
| 20 | Huochebang |
| 21 | Huolala |
| 22 | Ymm56 |
| 23 | Airbnb |
| 24 | Mayi |
| 25 | Tujia |
| 26 | Ziroom |
| 27 | Edaijia |
| 28 | UUPT |
| 29 | Imdada |
| 30 | Fengniao |
| 31 | Peisong |
| 32 | Ishansong |
| 33 | Iyuedan |
